# A novel superfamily containing the β-grasp fold involved in binding diverse soluble ligands

**DOI:** 10.1186/1745-6150-2-4

**Published:** 2007-01-24

**Authors:** A Maxwell Burroughs, S Balaji, Lakshminarayan M Iyer, L Aravind

**Affiliations:** 1 National Center for Biotechnology Information, National Library of Medicine, National Institutes of Health, Bethesda, MD 20894, USA; 2 Bioinformatics Program, Boston University, Boston, MA 02215, USA

## Abstract

**Background:**

Domains containing the β-grasp fold are utilized in a great diversity of physiological functions but their role, if any, in soluble or small molecule ligand recognition is poorly studied.

**Results:**

Using sensitive sequence and structure similarity searches we identify a novel superfamily containing the β-grasp fold. They are found in a diverse set of proteins that include the animal vitamin B12 uptake proteins transcobalamin and intrinsic factor, the bacterial polysaccharide export proteins, the competence DNA receptor ComEA, the cob(I)alamin generating enzyme PduS and the Nqo1 subunit of the respiratory electron transport chain. We present evidence that members of this superfamily are likely to bind a range of soluble ligands, including B12. There are two major clades within this superfamily, namely the transcobalamin-like clade and the Nqo1-like clade. The former clade is typified by an insert of a β-hairpin after the helix of the β-grasp fold, whereas the latter clade is characterized by an insert between strands 4 and 5 of the core fold.

**Conclusion:**

Members of both clades within this superfamily are predicted to interact with ligands in a similar spatial location, with their specific inserts playing a role in the process. Both clades are widely represented in bacteria suggesting that this superfamily was derived early in bacterial evolution. The animal lineage appears to have acquired the transcobalamin-like proteins from low GC Gram-positive bacteria, and this might be correlated with the emergence of the ability to utilize B12 produced by gut bacteria.

**Reviewers:**

This article was reviewed by Andrei Osterman, Igor Zhulin, and Arcady Mushegian.

## Background

The β-grasp fold (β-GF) was first recognized in ubiquitin and the immunoglobulin-binding (IG-binding) domains of Gram-positive cocci [1,2]. Since then it has come to be known as a widespread fold, utilized in proteins performing a great diversity of cellular functions. These include regulation of protein stability and signal transduction through the ubiquitin-conjugation system [3], RNA-protein interactions as seen in the TGS domain of tRNA synthetases [4], and adaptor functions involving protein-protein interactions as seen in the FERM module [5]. Additionally, standalone β-GF domain proteins ThiS/MoaD function as sulfur carriers in molybdopterin and thiamine biosynthesis [6] and the fold also provides an effective scaffold for binding iron-sulfur clusters in the case of the 2Fe-2S ferrodoxins involved in electron transport (see SCOP database [7]).

As part of our larger effort to understand the evolutionary and structural basis for the functional versatility of this widespread fold (for example see reference [8]) we were keen to determine if there were as yet uncharacterized representatives that might widen the functional horizon of the β-GF. In particular, we were interested in exploring the possibility of versions of the β-GF domains binding soluble ligands. Such a function was of interest because the presence of 2Fe-2S ferredoxins suggested that the β-GF domains could potentially provide a scaffold for binding a wider range of small molecules or other prosthetic groups. We accordingly investigated this further by applying a combination of sensitive structural comparisons and sequence profile analysis on members of the β-GF. As a result, we identify a novel domain superfamily with the β-GF fold and provide support that its members might be involved in binding different soluble ligands. We also study their genomic contexts, domain architectures and phyletic patterns to present evidence for their role in diverse metabolic networks, including those related to vitamin B12.

## Results and discussion

### Detection of sequence and structure relationships

**Figure 1:**
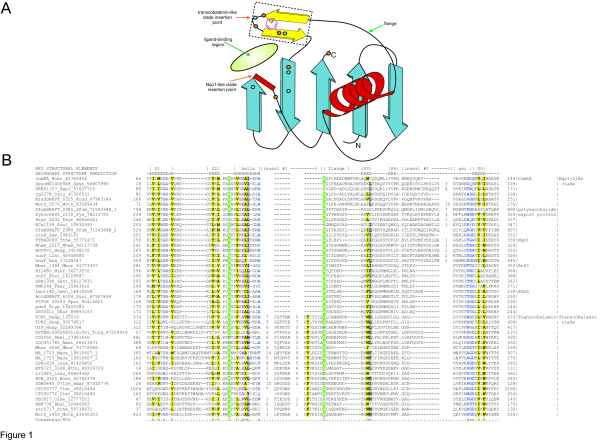
**Topology diagram of SLBB domain and multiple alignment of the SLBB superfamily**. (A) The five-stranded core (characteristic of all members of the βG-F) is shown with the helical face at the near side. β-strands are depicted as blue arrows, with the arrowhead at the C-terminus while the α-helix is shown in red. The two-strand insertion in the Transcobalamin-like clade is colored in yellow and enclosed in a dotted box. The insertion point for the Nqo1-like clade is marked by a red box. The approximate soluble ligand-binding spatial region is marked by a green oval. Residues known to contribute to cobalamin binding as derived from the crystal structure of Transcobalamin are shown as small circles. Orange circles indicate sidechain-mediated interactions while greenish blue circles indicate backbone or backbone and sidechain-mediated interactions. The conservation of an aromatic residue in Transcobalamin proteins is represented by a phenylalanine residue, rendered as a line drawing and colored purple. (B) Proteins are denoted by their gene names, species abbreviations, and gi numbers; demarcated by underscores. Amino acid residues are colored according to sidechain properties and degree of conservation within the alignment, set at 80% consensus. Consensus abbreviations are shown below the alignment. The secondary structure shown above the alignment is derived from the crystal structures of Transcobalamin and Nqo1 and secondary structure prediction programs. E and H denote β-strand and α-helix, respectively. Secondary structure elements conserved across the SLBB superfamily are labeled in the top line of the alignment. "Insert #1" refers to the Transcobalamin-like clade insert while "Insert #2" refers to the Nqo1-like clade insert. "asc" refers to the ascending connector between strands 4 and 5 often observed in the β-grasp fold. The consensus abbreviations and coloring scheme are as follows: h, hydrophobic residues (ACFILMVWY) shaded yellow; s, small residues (AGSVCDN) colored blue; p, polar residues (STEDKRNQHC) colored purple; and b, big residues (LIYERFQKMW) shaded gray. The conserved glycine residues characteristic of this superfamily are shaded light green and colored white. Species abbreviations are as follows: Aae: *Aquifex aeolicus*; Amel: *Apis mellifera*; Ana: *Nostoc *sp.; Bcer: *Bacillus cereus*; Bmar: *Blastopirellula marina*; Bthu: *Bacillus thuringiensis*; Cglu: *Corynebacterium glutamicum*; Ctet: *Clostridium tetani*; Dhaf: *Desulfitobacterium hafniense*; Dmel: *Drosophila melanogaster*; Ecol: *Escherichia coli*; Hinf: *Haemophilus influenzae*; Hsap: *Homo sapiens*; Krad: *Kineococcus radiotolerans*; Lint: *Leptospira interrogans*; Lmon: *Listeria monocytogenes*; Lreu: *Lactobacillus reuteri*; Lsak: *Lactobacillus sakei*; Mace: *Methanosarcina acetivorans*; Mbur: *Methanococcoides burtonii*; Moth: *Moorella thermoacetica*; Nham: *Nitrobacter hamburgensis*; Oihe: *Oceanobacillus iheyensis*; Ppro: *Photobacterium profundum*; Psyr: *Pseudomonas syringae*; Rbal: *Rhodopirellula baltica*; Sent: *Salmonella enterica*; Sepi: *Staphylococcus epidermidis*; Sfum: *Syntrophobacter fumaroxidans*; Spyo: *Streptococcus pyogenes*; Sthe: *Streptococcus thermophilus*; Styp: *Salmonella typhimurium*; Susi: *Solibacter usitatus*; Syn: *Synechococcus *sp.; Tmar: *Thermotoga maritima*; Tnig: *Tetraodon nigroviridis*; and Tthe: *Thermus thermophilus*.

To identify potential novel versions of the β-GF that bind soluble ligands, we initiated comprehensive structural comparisons with various previously characterized members of the fold (see β-grasp fold in SCOP database) using the DALI program. Several of these searches retrieved the C-terminal domain of the transcobalamin protein with significant Z-scores. For example, searches initiated with MoaD proteins (PDB: 1v8c, 1vjk) retrieved the C-terminal domain of transcobalamin (PDB: 2bbc) with Z-scores of ~7. Transcobalamin is an animal-specific protein that binds cobalamin (vitamin B12), and is involved in its uptake by animal cells [9]. Transcobalamin contains an N-terminal α/α toroid domain, and a C-terminal α/β domain [10] that corresponded to the β-GF domain recovered in the above searches. Further, DALI searches initiated with the C-terminal domain of transcobalamin recovered a diverse set of previously known β-GF domains such as MoaD (PDB:1vjk), YukD (PDB: 2bps) 2Fe-2S ferredoxin (PDB:1feh) and the middle domain of the Nqo1 subunit of the bacterial and mitochondrial NADPH-quinone oxidoreductase I complex (PDB: 2fug. S chain) with Z-scores in the range of 5–7. The structural alignments generated by these searches showed that the transcobalamin C-terminal domain aligns completely with all core structural elements of the β-GF, including a β-sheet of 5 strands and a helix between strands 2 and 3. However, the transcobalamin C-terminal domains are distinguished by the presence of a unique β-hairpin after the conserved helix of the β-GF (Figure 1A). The N-terminal α/α toroid domain and the C-terminal β-GF domain cooperate in ligand-binding by sandwiching a single B12 molecule between them [10]. Systematic searches for contacts between the B12 ligand and the C-terminal β-GF domain in transcobalamin showed that the unique insert plays a prominent role in binding the ligand by contributing several direct or solvent-mediated interactions [10]. Additional contacts with the ligand are also made by residues from the core β-GF such as those from strand 3, the end of strand 4 and the "ascending connector" between strand 4 and 5 (Figure 1A). These observations suggest that the C-terminal domain of transcobalamin represents a novel adaptation of the β-GF for small-molecule ligand interactions.

To better understand the diversity of this class of ligand-binding β-GF domains and their phyletic spread we initiated sequence profile and hidden Markov model (HMM) searches for homologs using PSI-BLAST and the HMMER package respectively. In addition to orthologs of transcobalamin, intrinsic factor and solo C-terminal domains from fishes, these searches retrieved numerous prokaryotic proteins, which were either present as stand-alone β-GF domains or in large multidomain proteins. For example, a search initiated with the β-GF domain of puffer fish transcobalamin (*Tetraodon nigroviridus*, gi: 47226456, region: 325–425) recovered closely related eukaryotic orthologs and paralogs fused to N-terminal α/α toroid modules (iteration 1), solo transcobalamin C-terminal domains with predicted signal peptides (e.g. XP_689937, *Danio rerio*, iteration 2, e-value: 3 × 10-12), and several prokaryotic proteins (e.g. BAC13773, *Oceanobacterium iheyensis*, iteration 3, e-value: 2 × 10-3). In order to exhaustively recover all divergent homologs, we conducted transitive searches with all above-detected members and also evaluated all hits below the threshold of PSI-BLAST searches for the presence of potentially homologous domains. We also prepared HMMs and PSSMs from the alignment of this region of all proteins recovered with significant expect-values (e < .01 with statistical correction for compositional bias) and used these to search all completely sequenced genomes. These searches consistently retrieved hits to multiple sequence repeats in a group of bacterial cell-surface/secreted sugar-binding proteins involved in polysaccharide export with significant e-values (e.g. *Hahella *periplasmic protein, HCH_02380 residues 852–990). Inclusion of polysaccharide export proteins in profiles for further searches additionally recovered the N-terminal region of the ComEA family of DNA uptake receptors of Gram positive bacteria (e.g. *Clostridium *ComEA, gi: 67874543, iteration 2, e = 10-6), PduS-like cobalamin reductases (e.g. *E. coli *cobalamin reductase iteration 7, e = 10-3), the middle domain of the 51 kD subunit (F chain) of the NADPH-quinone oxidoreductase complex I (Nqo1, E: 10-4, iteration 11) and the RnfC subunit of the oxidoreductases encoded by the bacterial Rnf operons [11](*Rhodobacter *RnfC, E: 10-6; iteration 14). This latter set of proteins was more similar to the homologous region recovered in the polysaccharide export proteins than to the transcobalamin C-terminal domain (Figure 1B). However, recovery of the middle domain of Nqo1 in sequence searches clearly confirms their relationship with transcobalamin C-terminal domains, because the former are also known, via structural analysis, to contain a similar β-GF domain [12] (See above and Additional file 1). This was additionally supported by separate secondary structure prediction for individual sub-groups with potentially homologous regions such as the ComEA N-terminal regions and the polysaccharide export proteins (Figure 1B).

We hereinafter refer to the homologous β-GF domains found in all these proteins as the Soluble-Ligand-Binding β-grasp (SLBB superfamily) as many members of this superfamily are known or predicted to bind soluble ligands (See below for further details).

### Sequence and structure features of the SLBB superfamily

A comprehensive multiple alignment for the SLBB superfamily (Figure 1B) was prepared by combining alignments for individual groups constructed using the T-Coffee program, based on the structural superposition of transcobalamin C-terminal domain (2bbc) and Nqo1 middle domain (2fug; chain S). Much of the conservation seen across the entire superfamily is in the form of hydrophobic residues forming the stabilizing core of the fold. However, there was a notable sequence feature in the form of two strongly conserved glycine residues, one in the turn leading into the horizontal flange preceding the third β-strand (Figure 1A) of the β-GF and the other immediately downstream of the second conserved β-strand (Figure 1). This conservation pattern is a unique feature of the SLBB superfamily that distinguishes them from all other previously characterized β-GF domains, supporting a common ancestry for this set of domains within the β-GF.

The alignment also helped us to classify the SLBB superfamily into several distinct families. The Transcobalamin C-terminal domain clade is unified by the presence of the above-described β-hairpin insert within the β-GF that plays an important role in contacting the ligand (Fig 1A, see Additional file 1). This β-hairpin contains a conserved hydrophobic position that makes a stacking interaction with the aromatic ring of the base in cobalamin. However, the rest of the sequence in this region is poorly conserved as most other interactions occur through backbone oxygen or nitrogen atoms [10] (Figure 1B). Within animals, insects and most vertebrates have a single ortholog of the B12 binding protein, whereas the mammals have three distinct versions, transcobalamin I, transcobalamin II and the intrinsic factor. Besides animals, members of this clade are widely represented in Low GC Gram-positive bacteria and planctomycetes and less frequently in the euryarchaea.

The Nqo1-like clade includes at least two distinct families: 1) the first includes the NADPH-quinone oxidoreductase complex I subunit Nqo1 (51 kD/F chain), the RnfC oxidoreductase subunit, and the PduS-like cobalamin reductases. 2) The second family contains polysaccharide export proteins and the DNA receptor ComEA. This clade is unified by the presence of a small, often α-helical insert, in the "connector arm" between the fourth and fifth strands of the domain (Figure 1, see Additional file 1). In some cases, such as the ComEA protein, the helical segment is followed by a low complexity region; suggesting the presence of a disordered, extended loop. These proteins are also characterized by an sGG motif (where 's' is any small residue) around the second conserved glycine of the superfamily (Figure 1B). The Nqo1 subunit of the classical NADPH-quinone oxidoreductase complex I is present in all major bacterial lineages with well-developed electron-transport chains, in most mitochondriate eukaryotic lineages, and very rarely in euryarchaea. The RnfC proteins are strongly conserved in γ-proteobacteria, but are also found in some representatives of Low GC Gram positive bacteria and the Bacteroidetes/Chlorobi assemblage. The PduS protein is restricted to the Low GC Gram-positive bacteria and certain γ and δ proteobacteria. The ComEA proteins and polysaccharide export proteins show a nearly mutually exclusive complementary distribution. The ComEA family is chiefly present in Low GC Gram-positive bacteria and actinobacteria, whereas the polysaccharide export family is more widespread and widely present in proteobacteria, cyanobacteria, acidobacteria, planctomycetes, bacteroidetes/chlorobi, and more sporadically in a few other groups.

While the interaction between B12 and the transcobalamin-like SLBB domain involves the unique β-hairpin insert, key contacts are also contributed by the core fold (See above, Figure 1A), and in general the position of the bound ligand is comparable to that of the bound metal-sulfur cluster in the β-GF ferredoxins. The Nqo1-like clade shows its distinctive innovation in the region between strands 4 and 5, which also corresponds to the same general spatial location where the ligands are bound in the transcobalamin-like clade and β-GF ferredoxins (Figure 1B). This indicates that the structural innovation specific to the Nqo1-like clade might also be involved in binding a ligand at a similar position. This spatial location might thus represent a common site for soluble ligand interactions in the β-GF that is distinct from the C-terminal tail and the opposite protein surface that is key to the functional interaction of sulfur carriers like ThiS and MoaD and the ubiquitin-like proteins [13].

### Contextual associations and inferences of possible functions for the SLBB

To investigate the functional diversification of the SLBB fold we used contextual analysis, which often provides insights into biochemical functions of poorly characterized protein domains or genes. Contextual analysis utilizes the information gleaned from the association of uncharacterized domains with other domains of known function and the tendency of genes whose products functionally interact to associate in conserved gene neighborhoods or predicted operons. [14-16].

**Figure 2:**
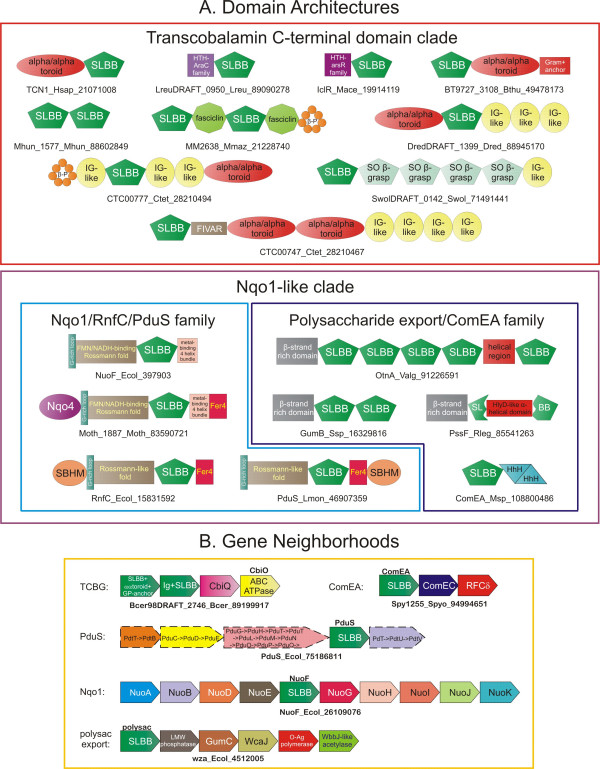
**Domain architectures and conserved gene neighborhoods of the SLBB superfamily**. (A) Representative architectures are grouped according to family/clade type and labeled by gene name, organism abbreviation, and gi number; demarcated by underscores. Individual domains in architectures are depicted as colored polygons. (B) A sampling of conserved gene neighborhoods found in association with the SLBB domain, with genes depicted as boxed arrows. The SLBB members of the depicted gene neighborhoods are labeled below by gene name, organism abbreviation, and gi number; demarcated by underscores. The family/clade name of the SLBB domain is given to the left in each architecture. Names are given at the top of genes in neighborhoods, where appropriate. The large PduS operon is broken into commonly-observed gene clusters; each boxed arrow enclosed by a dotted line represents such a cluster. ComEA proteins are always in the vicinity of the two-domain ComEC protein that has an integral membrane domain predicted to form a pore through which the DNA is transported into the cell and a metallo-β-lactamase-like domain that may serve as a DNA nuclease during the uptake DNA from the outer cell wall. Additional organism abbreviations not given in Figure 1: *Mhun*, *Methanospirillum hungatei*; *Mmaz*, *Methanosarcina mazei*; *Dred*, *Desulfotomaculum reducens*; *Swol*, *Syntrophomonas wolfei*; *Valg*, *Vibrio alginolyticus*; *Rleg*, *Rhizobium leguminosarum*; *and Msp*, *Mycobacterium sp. MCS*. Additional abbreviations: SO, Sulfite Oxidoreductase; β-P, β-propeller; Fer4, 4Fe-S ferrodoxin; HTH, Helix-Turn-Helix; HhH, Helix-hairpin-Helix; RFCδ, RFC clamp loader subunit; LMW, low-molecular weight; and O-Ag, O-antigen.

Most members of the transcobalamin C-terminal domain clade of the SLBB superfamily contain signal peptides, and several also contain the C-terminal Gram-positive anchor motif [17], suggesting that they are secreted or cell-surface proteins. A common domain architecture in this clade encountered in both eukaryotes and bacteria is the fusion of the SLBB to an α/α toroid domain. In bacteria the toroid may be present either N-terminal (e.g. *Desulfotomaculum*, gi: 88945170) or C-terminal (e.g. *Bacillus*, gi: 42782379) to the SLBB (Figure 2A). As the central cavity formed by the α/α toroid in transcobalamin plays a major role in binding B12 [10], it is likely that the two domains cooperate in binding B12 in all these proteins. Additional architectures include fusions to domains typically found in extracellular proteins, such as one or more immunoglobulin-fold domains (e.g. *Archaeoglobus*; gi: 11498993 and *Moorella*; gi: 83590303), the FIVAR (Pfam entry: PF07554) sugar-binding domain (*Clostridium*, gi: 28210467), the fasciclin domain (*Methanosarcina*; gi: 21228740) and a β-propeller domain (*Clostridium*, gi: 28210494). Given that many of these domains are often involved in interactions with polysaccharides, they might play a role in tethering these proteins to the cell surface by binding peptidoglycan or capsular polysaccharides [18-23]. Often these multi-domain SLBB proteins occur in conserved operons that might additionally code a second paralogous extracellular SLBB protein (Figure 2). This might imply that different extracellular SLBB proteins interact together to form protein complexes on the cell surface. Interestingly, an analysis of the B12 biosynthesis pathways of all the bacteria that possess proteins with such SLBB domains showed they usually lacked key biosynthetic enzymes for B12. Furthermore, these SLBB proteins are generally encoded by predicted operons that also contain genes for CbiO-like ABC ATPase and the CbiQ-like integral membrane protein implicated in cobalt transport [24]. These observations suggest that the primary role of this clade of SLBB proteins might be to scavenge B12 or its precursors from the environment. As the archaea which contain these SLBB proteins often possess an anaerobic B12 synthesis pathway, it is possible that these might instead be involved in scavenging a distinct metabolite. In *Syntrophomonas *the SLBB domain is found in putative extracellular enzymes fused to sulfite oxidase-like molybdopterin cofactor binding domain (e.g. gi: 71491441) [25]. It is likely that in these proteins the SLBB provides a B12 cofactor that might be required by these enzymes.

Intracellular versions of the transcobalamin-like clade show fusions of the SLBB domain with two distinct winged HTH domains, namely those of the ArsR-like (e.g. gi:72395507, *Methanosarcina*) and AraC-like families (E.g. gi: 86604362, *Lactobacillus*) (Figure 2A). These proteins probably function as one-component transcription factors that respond to concentration of B12, its precursors or some other as yet unknown soluble ligands.

In the Nqo1-like clade, polysaccharide export proteins are predicted to be secreted or periplasmic proteins and contain an absolutely conserved N-terminal β-strand-rich domain followed by 1–8 repeats of the SLBB domain (Figure 2A). They appear to be part of a larger complex that is involved in transport of polysaccharides to the cell surface and are believe to associate with the outer membrane and periplasmic space in proteobacteria [26]. Conserved gene-neighborhoods that encode these proteins are populated with proteins involved in the biosynthesis and modification of sugars or polysaccharides, which is consistent with their role in polysaccharide export (Figure 2). The related ComEA proteins of Gram-positive bacteria also contain a signal peptide followed by an N-terminal SLBB domain that is always fused to a pair of DNA-binding Helix-hairpin-Helix domains at their C-terminus. This is consistent with the role of the ComEA protein as a non-specific DNA receptor in the transformation competence mechanism of Gram-positive bacteria [27,28]. Prior studies suggest that this DNA receptor may be linked to the cell surface via the N-terminal region spanning the SLBB domain [27]. Taken together these observations suggest that the SLBB domain in these proteins is likely to be critical for interaction with cell polysaccharides and/or sugars of the peptidoglycan. The complementary phyletic distribution of ComEA and polysaccharide export proteins is strongly correlated with the presence or the absence of the specialized Gram-positive cell wall (See above). This suggests that they probably diverged from a common ancestral polysaccharide/sugar-binding domain that was originally involved in uptake or extrusion of large molecules at the cell surface.

The remaining three groups of proteins, namely NqoI, RnfC and PduS, within the Nqo1-like clade of the SLBB superfamily share a common architectural core consisting of a fusion between an N-terminal Rossmannoid domain and an SLBB domain. Unlike classical Rossmann fold domains of oxidoreductases with 5–7 strands, the Rossmannoid domain of these proteins has a 4-stranded core in a 3214 order [12] coupled to a N-terminal two-stranded hairpin contributed by a module similar to the BBMs of RNA polymerases [29]. The SLBB and this Rossmannoid domain are additionally combined to a variety of other domains in NqoI, RnfC and PduS proteins. The most common fusions seen in all three groups of proteins are those to 4Fe-S ferredoxin domains that flank the above two-domain core. The NqoI family also might contain a C-terminal tetrahelical bundle with an up-and-down topology that coordinates an Fe-S cluster via conserved cysteine residues, which in addition to the 4Fe-S ferredoxin is likely to provide an additional redox center for electron transport [12]. A biotin/lipoate carrier-like Sandwich Barrel Hybrid Motif (SBHM) domain [29] is found respectively at the C- and N-termini of members of PduS and RnfC families.

The roles of these versions of the SLBB remain enigmatic; however there is evidence that the PduS protein might bind soluble ligands. The PduS gene typically belongs to a large mobile operon coding proteins required for the biogenesis of carboxysome/polyhedral bodies, which contains enzymes involved in propanediol degradation. The PduS has been shown to strongly bind cob(I)alamin and was characterized as a bifunctional cob(II)alamin and hydroxycobalamin (cob(III)alamin) reductase catalyzing the formation of cob(I)alamin. Cob(I)alamin is the immediate precursor of Ado-cobalamin, which serves as an essential coenzyme for the diol dehydratase in degradation of 1,2-propanediol [30,31]. It is likely that the SLBB domain in PduS, like that in transcobalamin, binds cob(I)alamin or HO-cobalamin, while the N-terminal Rossmanoid domain binds the flavin nucleotide cofactor for the redox reaction. Such a function is also supported by the observation that cob(I)alamin is highly reactive and needs to be shielded from the environment [30]. The role of the fused SBHM domain seen in PduS proteins is less clear. However, given that the SBHM domain carries covalently associated ligands such as biotin/lipoate [32,33], it might similarly carry cofactor ligands or intermediates in propanediol degradation such as 1,2-propanediol-1-yl radical [34]. There is currently no evidence for a soluble ligand interacting with the related SLBB domain in the RnfC and NqoI. Nevertheless, crystal structures indicate an exposed location for the SLBB domains in these proteins, allowing the possibility that they might be allosterically regulated by hitherto unknown ligands interacting with this domain.

## Evolutionary history of the SLBB domain and general conclusions

The phyletic and domain-architecture distributions show that the SLBB superfamily is well-represented and has diversified across the entire bacteria superkingdom. Their sporadic presence in archaea and the stronger affinity of the different eukaryotic versions to their bacterial counterparts suggests that the SLBB superfamily was derived early in evolution of bacteria, most probably from one of the many ancient β-grasp fold domains. The two major families in the NqoI-like clade appear to have a pan-bacterial distribution suggesting that this clade had already differentiated into versions associated with cell wall related functions (ComEA-Polysaccharide export protein family) and intracellular oxido-reductase related functions (Nqo1, RnfC and PduS). Of the latter group the NqoI protein of the respiratory complex-I is seen across bacteria and was transferred to the eukaryotic lineage during the primary endosymbiotic event that generated the eukaryotic cell with mitochondria. RnfC and PduS proteins have more restricted phyletic distributions and are likely to be late derivatives of the more ancient Nqo1 lineage. The transcobalamin C-terminal clade is very divergent in sequence and appears to be a lineage-specific innovation in Gram positive bacteria that was recruited specifically for transporting extracellular B12-like cofactors or their precursors. Subsequently, a specific version that combined the α/α toroid domain with the SLBB domain appears to have been laterally transferred to the animal lineage early in its evolution. This event probably conferred on animals the ability to directly absorb cobalamin synthesized by bacteria in the gut. In parallel, there appear to have been sporadic transfers of large extracellular multidomain versions as well as intracellular versions fused to DNA-binding HTH domains to certain euryarchaeal lineages.

In this context, it is of interest to note that the discovery of soluble ligand binding versions of the β-GF points to a noteworthy structure-function analogy with the RNA-recognition motif (RRM)-like fold. In functional terms, the RRM-like fold has long been known to bind a range of soluble ligands such as amino acids, sugars and co-factors. Notable examples of these include the ACT domain superfamily and the amino acid-binding domain of the LRP-like transcription factors [20,35,36]. Both folds also provide scaffolds for iron-sulfur clusters, (4Fe-4S ferrodoxins in the case of the RRM-like fold [37]) and are also involved in RNA-binding, as well as adaptor functions related to protein-protein interactions [32,33]. These functional analogies in turn might be related to certain general organizational similarities seen in the two domains: like the β-GF domain, the RRM-like fold domain is also a relatively small domain with an asymmetric two-layered structure. One surface of the core sheets is partially obscured by helical segments in both these folds, whereas the other is largely left exposed (see SCOP database [7]). Further study of these functional analogies might throw light on whether there exist certain general structural principles that have affected the recruitment of certain small ancient domains in similar contexts.

In conclusion, we show that the β-GF domains found in transcobalamin, polysaccharide export proteins, ComEA, PduS, and RnfC and Nqo1-like oxidoreductases define a novel superfamily, several of which might interact with different soluble ligands. This investigation provides the possible evolutionary scenario for the origin of the vitamin B12 uptake in animals via transcobalamin and intrinsic factor. It also provides leads for new investigations into B12 metabolism in bacteria and other aspects of protein-ligand interaction in competence, cell-surface biochemistry, and respiratory electron transfer.

## Methods

Searches of the PDB database with query structures were conducted using the DALI program [38]. Structural visualization and manipulations were performed using the Swiss-PDB viewer program [39]. Sensitive profile searches were conducted using the PSI-BLAST [40] and HMMER programs [41]. PSI-BLAST searches were performed against the nonredundant (NR) database of protein sequences (National Center for Biotechnology Information [NCBI], NIH, Bethesda, MA, USA), with either a single sequence or an alignment used as the query, with a default profile inclusion expectation (e) value threshold of 0.01 (unless specified otherwise), and was iterated until convergence. All sequences collected in these searches are made available in Additional file 2. The library of profiles for various domains was prepared by extracting all alignments from the PFAM database [42] and updating them by adding new members from the NR database. These updated alignments were then used to make HMMs with the HMMER package or PSSMs with PSI-BLAST. For all searches involving membrane-spanning domains we used a statistical correction for compositional bias to reduce false positives due to the general hydrophobicity of these proteins [43]. Signal peptide and transmembrane helices were predicted using the SignalP [44] and TMHMM programs [45]. Multiple alignments were constructed using the T_Coffee [46] and MUSCLE programs [47] followed by manual adjustments based on PSI-BLAST results. Protein secondary structure was predicted using a multiple alignment as the input for the JPRED program [48], with information extracted from a PSSM, HMM, and the seed alignment itself. Similarity-based clustering of proteins was carried out using the BLASTCLUST program [49]. Gene neighborhoods were determined using a custom script that uses completely sequenced genomes or whole genome shotgun sequences to derive a table of gene neighbors for a query gene. The BLASTCLUST program was then used to cluster the proteins sequences in the neighborhoods and establish conserved co-occurring genes. The KEGG database was used to identify key components of the B12 synthesis pathway [50]. Automation of all large-scale sequence analysis procedures were carried out using the in-house TASS package (Anantharaman V, Balaji S, Aravind L; unpublished), which operates similar to the previously published SEALS package [51].

## Reviewers' comments

### Reviewer's report 1

#### Andrei Osterman, Burnham Institute for Medical Research

The manuscript "A novel superfamily with the β-grasp fold involved in binding diverse soluble ligands" by A. M. Burroughs is an excellent and insightful comparative genomics study of a very interesting domain family broadly conserved across all kingdoms of Life. The authors took on a very challenging task of classifying multiple representatives of this superfamily on the edge of detectable homology. Such a task is particularly daunting for non-enzymatic proteins, where the divergence of sequence driven by adaptation to new biological tasks and contexts is much more pronounced than for enzymes. This study tracks down a complex evolution of the family and reveals an apparent functional theme, binding of small molecule ligands, such as B12 and its analogs, that "runs in the family" from bacteria to mammals. In addition to sensitive and sophisticated homology-based methods, the authors broadly used genome context analysis, which was one of the key success factors in pursuing this challenge. Of particular interest is the observation that in course of a fascinating evolutionary reshuffling, not only the ligand binding specificity, but the actual functional context of this domain could have changed on a number of occasions. One of the most remarkable events is a proposed recruitment of this domain into the context of transcriptional regulation in some bacteria. The latter theme of recruiting former enzymes and, as we see now, other types of proteins with natural affinity to certain ligands, is being recognized as one of the most important strategies of "natural engineering" of effector-binding domains in transcriptional regulators. Although this bioinformatics analysis alone does not allow to precisely identifying specific functions for all SLBB subfamilies, it certainly provides a perfect starting point for many case studies driven by specific research interests of various experimental groups. This constitutes a broad impact of this paper, which goes beyond the innovative bioinformatics methodology and obvious implications in the field of domain classification and evolution. The paper is written very clearly, with sufficient details of methods and key results and with helpful illustrations. The Supplementary material providing the entire list of SLBB domain superfamily including the information about their genome context, is highly valuable. This reviewer strongly supports the publication of this paper "as is" in Biology Direct, a perfect home for this wonderful study.

#### Author response

*We appreciate the comments, and particularly would like to emphasize that we feel the findings in this paper can be used as starting points for experimental work that will contribute significantly to the study of several important biological pathways*.

### Reviewer's report 2

#### Igor Zhulin, University of Tennessee

In this study, the authors report a novel domain superfamily within the β-grasp fold. The predicted distinct property of this domain is binding diverse soluble ligands, thus it is termed Soluble-Ligand-Binding β-grasp or SLBB. Motivation for this work came from expectation (but lack of factual knowledge) that some members of the ubiquitous β-grasp fold are involved in binding small ligands.

First, the authors searched the PDB database with known structures to retrieve related structures and then used an array of sensitive sequence-based searches (hidden-Markov-model- or position-specific-scoring-matrix-based) to identify remote homologs. Technically, it is very well done and described in sufficient detail. The authors took advantage of available structures to guide editing of the multiple alignment, which in turn allowed them to associate conserved positions with the structural features.

Biological function prediction came primarily from the contextual analysis, which was quite thorough. The most interesting prediction is that the primary role of one of the two SLBB clades is binding vitamin B12 and its precursors.

Overall, this is an interesting, well-executed study and defining novel domain families and assigning potential biological functions is very important. I would like to add that it is also important to deposit the newly described domain to leading domain databases, such as Pfam. I have not seen plans for doing so in this study. This reviewer is guilty of not always doing so either, but still it should be a rule rather than exception. Perhaps, two version of SLBB could be produced capturing characteristics of the two clades.

#### Author response

*We could not agree more regarding the importance of domain databases, and we will be submitting two versions of the SLBB to Pfam*.

##### Major concern

Essentially, there is only one major concern with respect to generalization of biological function prediction. I think the members of the domain clade are divergent enough to worry about B12 and its precursors being the only or even the main ligand. The statement in the abstract seems to address this issue – the authors rightly indicate that "members of this superfamily are likely to bind a range of soluble ligands, including B12"; however in the text it becomes more stringent. For instance, on page 3, when describing SLBB fusions with HTH domains in transcription factors, it is suggested that these proteins "respond to B12 or its precursors". I would suggest toning down the claim that all members of the transcobalamin clade bind exclusively B12 and its precursors. Perhaps, the authors did not intend to make it sounds like that, but it does.

#### Author response

*We do admit that there might be a greater diversity of bound ligands, rather than B12 alone. Only in the cases where there was contextual evidence for involvement in B12 metabolism do we suggest it as a most likely ligand. Following the suggestion of the reviewer, we have altered the wording in the text to clarify the possibility of greater diversity in terms of bound ligands, especially with the HTH-fused versions*.

### Reviewer's report 3

#### Arcady Mushegian, Stowers Institute, Kansas City, USA

I have no concerns about the main observation of the extended beta-grasp SLBB superfamily, or about functional inferences from the contextual analysis of genes that contain various versions of this domain. I think, however, that the analogy with the RRM fold early in the manuscript is a distraction at that point of the authors' reasoning. Generally, I suppose that this type of domain/fold recognition work may benefit from being more logical and less chronological: that is, even if analogy with RRM played a role in authors' own thinking about the SLBB superfamily, I want to first focus on what has been actually observed. On the other hand, I would like to see a discussion of RRM and other analogous folds at the end of the manuscript, when it could serve a more useful purpose of establishing some trends in evolution (do we see lots of small ligand-binding domains evolving from nucleic-acid binding domains? How about the other way around? And so on).

#### Author's response

*We admit this might provide a better flow for the article. Accordingly, we have now shifted the discussion of the parallels between the β-grasp and RRM folds to the Results and Discussion section*.

## Authors' contributions

LA and SB made the initial discovery reported here. The complete sequence and structure analysis and preparation of figures and supplementary material was performed by AMB, LMI and SB. The paper was written by AMB and LA.

## Supplementary Material

Additional file 1 **Cartoon representations of SLBB domains**. File 1 contains cartoon representations of the transcobalamin and the Nqo1 middle domain showing the structural similarity and innovations associated with the potential binding site for soluble ligands. Click here for file

Additional file 2 **Comprehensive list of proteins of the SLBB superfamily, their gene neighborhoods, and domain architectures**. Alignments of the SLBB domain will be submitted to the Pfam domain database [39]. Click here for file

## References

[B1] Kraulis PJ (1991). Similarity of protein G and ubiquitin. Science.

[B2] Murzin AG (1992). Familiar strangers. Nature.

[B3] Hershko A, Ciechanover A (1998). The ubiquitin system. Annu Rev Biochem.

[B4] Wolf YI, Aravind L, Grishin NV, Koonin EV (1999). Evolution of aminoacyl-tRNA synthetases--analysis of unique domain architectures and phylogenetic trees reveals a complex history of horizontal gene transfer events. Genome Res.

[B5] Chishti AH, Kim AC, Marfatia SM, Lutchman M, Hanspal M, Jindal H, Liu SC, Low PS, Rouleau GA, Mohandas N, Chasis JA, Conboy JG, Gascard P, Takakuwa Y, Huang SC, Benz EJ, Bretscher A, Fehon RG, Gusella JF, Ramesh V, Solomon F, Marchesi VT, Tsukita S, Tsukita S, Hoover KB (1998). The FERM domain: a unique module involved in the linkage of cytoplasmic proteins to the membrane. Trends Biochem Sci.

[B6] Rudolph MJ, Wuebbens MM, Rajagopalan KV, Schindelin H (2001). Crystal structure of molybdopterin synthase and its evolutionary relationship to ubiquitin activation. Nat Struct Biol.

[B7] SCOP database.

[B8] Iyer LM, Burroughs AM, Aravind L (2006). The prokaryotic antecedents of the ubiquitin-signaling system and the early evolution of ubiquitin-like beta-grasp domains. Genome Biol.

[B9] Moestrup SK (2006). New insights into carrier binding and epithelial uptake of the erythropoietic nutrients cobalamin and folate. Curr Opin Hematol.

[B10] Wuerges J, Garau G, Geremia S, Fedosov SN, Petersen TE, Randaccio L (2006). Structural basis for mammalian vitamin B12 transport by transcobalamin. Proc Natl Acad Sci U S A.

[B11] Schmehl M, Jahn A, Meyer zu Vilsendorf A, Hennecke S, Masepohl B, Schuppler M, Marxer M, Oelze J, Klipp W (1993). Identification of a new class of nitrogen fixation genes in Rhodobacter capsulatus: a putative membrane complex involved in electron transport to nitrogenase. Mol Gen Genet.

[B12] Sazanov LA, Hinchliffe P (2006). Structure of the hydrophilic domain of respiratory complex I from Thermus thermophilus. Science.

[B13] Mossessova E, Lima CD (2000). Ulp1-SUMO crystal structure and genetic analysis reveal conserved interactions and a regulatory element essential for cell growth in yeast. Mol Cell.

[B14] Overbeek R, Fonstein M, D'Souza M, Pusch GD, Maltsev N (1999). The use of gene clusters to infer functional coupling. Proc Natl Acad Sci U S A.

[B15] Huynen M, Snel B, Lathe W, Bork P (2000). Predicting protein function by genomic context: quantitative evaluation and qualitative inferences. Genome Res.

[B16] Wolf YI, Rogozin IB, Kondrashov AS, Koonin EV (2001). Genome alignment, evolution of prokaryotic genome organization and prediction of gene function using genomic context. Genome Res.

[B17] Fischetti VA, Pancholi V, Schneewind O (1990). Conservation of a hexapeptide sequence in the anchor region of surface proteins from gram-positive cocci. Mol Microbiol.

[B18] Williams RJ, Henderson B, Sharp LJ, Nair SP (2002). Identification of a fibronectin-binding protein from Staphylococcus epidermidis. Infect Immun.

[B19] Aravind L, Anantharaman V, Iyer LM (2003). Evolutionary connections between bacterial and eukaryotic signaling systems: a genomic perspective. Curr Opin Microbiol.

[B20] Aravind L, Koonin EV (1999). Gleaning non-trivial structural, functional and evolutionary information about proteins by iterative database searches. J Mol Biol.

[B21] Hofmann BE, Bender H, Schulz GE (1989). Three-dimensional structure of cyclodextrin glycosyltransferase from Bacillus circulans at 3.4 A resolution. J Mol Biol.

[B22] Ulstrup JC, Jeansson S, Wiker HG, Harboe M (1995). Relationship of secretion pattern and MPB70 homology with osteoblast-specific factor 2 to osteitis following Mycobacterium bovis BCG vaccination. Infect Immun.

[B23] Clout NJ, Tisi D, Hohenester E (2003). Novel fold revealed by the structure of a FAS1 domain pair from the insect cell adhesion molecule fasciclin I. Structure.

[B24] Rodionov DA, Hebbeln P, Gelfand MS, Eitinger T (2006). Comparative and functional genomic analysis of prokaryotic nickel and cobalt uptake transporters: evidence for a novel group of ATP-binding cassette transporters. J Bacteriol.

[B25] Kisker C, Schindelin H, Pacheco A, Wehbi WA, Garrett RM, Rajagopalan KV, Enemark JH, Rees DC (1997). Molecular basis of sulfite oxidase deficiency from the structure of sulfite oxidase. Cell.

[B26] McNulty C, Thompson J, Barrett B, Lord L, Andersen C, Roberts IS (2006). The cell surface expression of group 2 capsular polysaccharides in Escherichia coli: the role of KpsD, RhsA and a multi-protein complex at the pole of the cell. Mol Microbiol.

[B27] Inamine GS, Dubnau D (1995). ComEA, a Bacillus subtilis integral membrane protein required for genetic transformation, is needed for both DNA binding and transport. J Bacteriol.

[B28] Provvedi R, Dubnau D (1999). ComEA is a DNA receptor for transformation of competent Bacillus subtilis. Mol Microbiol.

[B29] Iyer LM, Koonin EV, Aravind L (2003). Evolutionary connection between the catalytic subunits of DNA-dependent RNA polymerases and eukaryotic RNA-dependent RNA polymerases and the origin of RNA polymerases. BMC Struct Biol.

[B30] Sampson EM, Johnson CL, Bobik TA (2005). Biochemical evidence that the pduS gene encodes a bifunctional cobalamin reductase. Microbiology.

[B31] Bobik TA, Havemann GD, Busch RJ, Williams DS, Aldrich HC (1999). The propanediol utilization (pdu) operon of Salmonella enterica serovar Typhimurium LT2 includes genes necessary for formation of polyhedral organelles involved in coenzyme B(12)-dependent 1, 2-propanediol degradation. J Bacteriol.

[B32] Perham RN (2000). Swinging arms and swinging domains in multifunctional enzymes: catalytic machines for multistep reactions. Annu Rev Biochem.

[B33] Anantharaman V, Koonin EV, Aravind L (2001). Regulatory potential, phyletic distribution and evolution of ancient, intracellular small-molecule-binding domains. J Mol Biol.

[B34] Yamanishi M, Ide H, Murakami Y, Toraya T (2005). Identification of the 1,2-propanediol-1-yl radical as an intermediate in adenosylcobalamin-dependent diol dehydratase reaction. Biochemistry.

[B35] Leonard PM, Smits SH, Sedelnikova SE, Brinkman AB, de Vos WM, van der Oost J, Rice DW, Rafferty JB (2001). Crystal structure of the Lrp-like transcriptional regulator from the archaeon Pyrococcus furiosus. Embo J.

[B36] Chipman DM, Shaanan B (2001). The ACT domain family. Curr Opin Struct Biol.

[B37] Sticht H, Rosch P (1998). The structure of iron-sulfur proteins. Prog Biophys Mol Biol.

[B38] Holm L, Sander C (1995). Dali: a network tool for protein structure comparison. Trends Biochem Sci.

[B39] Guex N, Peitsch MC (1997). SWISS-MODEL and the Swiss-PdbViewer: an environment for comparative protein modeling. Electrophoresis.

[B40] Altschul SF, Madden TL, Schaffer AA, Zhang J, Zhang Z, Miller W, Lipman DJ (1997). Gapped BLAST and PSI-BLAST: a new generation of protein database search programs. Nucleic Acids Res.

[B41] Eddy SR (1998). Profile hidden Markov models. Bioinformatics.

[B42] Finn RD, Mistry J, Schuster-Bockler B, Griffiths-Jones S, Hollich V, Lassmann T, Moxon S, Marshall M, Khanna A, Durbin R, Eddy SR, Sonnhammer EL, Bateman A (2006). Pfam: clans, web tools and services. Nucleic Acids Res.

[B43] Schaffer AA, Aravind L, Madden TL, Shavirin S, Spouge JL, Wolf YI, Koonin EV, Altschul SF (2001). Improving the accuracy of PSI-BLAST protein database searches with composition-based statistics and other refinements. Nucleic Acids Res.

[B44] Bendtsen JD, Nielsen H, von Heijne G, Brunak S (2004). Improved prediction of signal peptides: SignalP 3.0. J Mol Biol.

[B45] Krogh A, Larsson B, von Heijne G, Sonnhammer EL (2001). Predicting transmembrane protein topology with a hidden Markov model: application to complete genomes. J Mol Biol.

[B46] Notredame C, Higgins DG, Heringa J (2000). T-Coffee: A novel method for fast and accurate multiple sequence alignment. J Mol Biol.

[B47] Edgar RC (2004). MUSCLE: a multiple sequence alignment method with reduced time and space complexity. BMC Bioinformatics.

[B48] Cuff JA, Clamp ME, Siddiqui AS, Finlay M, Barton GJ (1998). JPred: a consensus secondary structure prediction server. Bioinformatics.

[B49] BLASTCLUST program.

[B50] Kanehisa M, Goto S, Hattori M, Aoki-Kinoshita KF, Itoh M, Kawashima S, Katayama T, Araki M, Hirakawa M (2006). From genomics to chemical genomics: new developments in KEGG. Nucleic Acids Res.

[B51] Walker DR, Koonin EV (1997). SEALS: a system for easy analysis of lots of sequences. Proc Int Conf Intell Syst Mol Biol.

