# Supplementary figures and images for: A novel superfamily containing the β-grasp fold involved in binding diverse soluble ligands
Source: Biol Direct. 2007 Jan 24;2:4. doi: 10.1186/1745-6150-2-4 (PMC1796856; doi:10.1186/1745-6150-2-4)

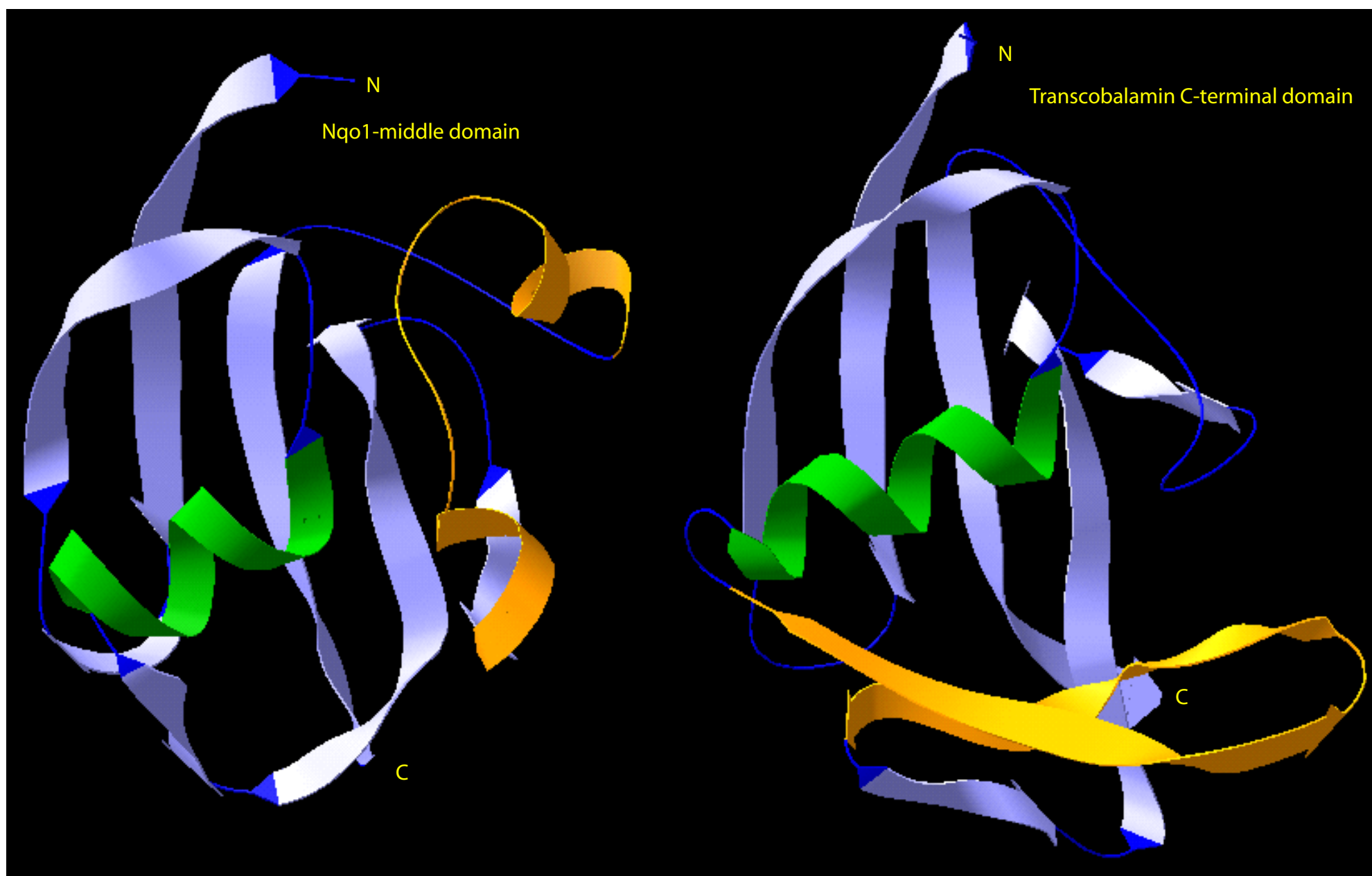

Supplement: Additional file 1 — Cartoon representations of SLBB domains. File 1 contains cartoon representations of the transcobalamin and the Nqo1 middle domain showing the structural similarity and innovations associated with the potential binding site for soluble ligands. [file 1745-6150-2-4-S1.pdf]
